# Accident to Mr. A. W. Faire, Deputy-Lieutenant of Leicestershire

**Published:** 1916-12-30

**Authors:** 


*To the Editor of* THE HOSPITAL.

### Accident to Mr. A. W. Faire, Deputy-Lieutenant of Leicestershire.

MISS ELLEN M. GRIMSLEY, Secretary, Leicestershire Territorial Association, writes on December 23, 1916 :

*To the Editor of* THE HOSPITAL.

SIR,—I am enclosing you a copy of a letter received from Mr. Harry Johnson, house governor and secretary to the Leicester Royal Infirmary. I may say that the honours referred to are a Deputy-Lieutenancy of Leicestershire and a Knight of Grace of the Order of St. John. Mr. Faire unfortunately met with an accident a few days ago, when he fell and broke his right arm. He is an inmate of the 1/5th Northern General Hospital, where he is, of course, receiving every attention.

The following is a copy of Mr. Johnson's letter to Mr. Faire :—

On the motion of the chairman, Mr. T. Fielding-Johnson, seconded by Colonel C. J. Bond, the chairman and vice-chairman of the board of this institution, a resolution was passed at the meeting of the governors on December 20 congratulating you most heartily on the honours so recently conferred upon you, and commiserating with you in the recent accident which you all too unfortunately sustained. The governors are mindful of the very great help which you have indirectly given to the institution in connection with the transport arrangements of wounded soldiers, and they sincerely hope that you will be speedily restored to your usual health, and that your accident may leave behind no unhappy results.

